# A mixed-methods national study investigating key challenges in learning English as a foreign language: A Chinese college student perspective

**DOI:** 10.3389/fpsyg.2022.1035819

**Published:** 2022-12-16

**Authors:** Jinyan Huang, Junfei Li, Tiantian Shu, Yongfang Zhang

**Affiliations:** ^1^School of Education, Jiangsu University, Zhenjiang, China; ^2^School of Foreign Language, Shanghai Dianji University, Shanghai, China; ^3^School of Education, Yan’an University, Yan’an, Shaanxi, China

**Keywords:** English as a foreign language (EFL), learning challenges, English listening, speaking, reading, writing skills, Chinese college students, Teaching English to Speakers of Other Languages (TESOL)

## Abstract

**Introduction:**

This mixed-methods national study has a two-fold purpose: (a) to invite Chinese college students to rate their overall difficulties in the eight areas of English learning, i.e., listening, speaking, reading, writing, pronunciation, grammar, vocabulary, and culture; and (b) to invite them to identify their key challenges in learning EFL. Specifically, the following three research questions were asked: (a) what are their overall difficulties in the eight areas of learning EFL? (b) Are there significant differences in rating their overall difficulties in these eight areas across the demographic variables of gender (i.e., male versus female participants) and subject discipline (arts versus sciences versus English subject disciplines participants)? And (c) what are their key challenges in learning EFL?

**Methods:**

The participants included a sample of 1,525 freshmen and sophomore students currently studying at seven universities across China. The instrument was a survey that consisted of a five-point Likert scale self-evaluation form and a major essay question addressing their key challenges. The qualitative data can help to probe deeply into the research setting to obtain in-depth understandings about Chinese college students’ English learning; and therefore, they can greatly enhance the quantitative data.

**Results and discussion:**

The quantitative results indicated that listening, speaking, and writing were their three key difficult areas in EFL learning; further, students’ subject discipline (arts versus sciences verse English subject disciplines) had significant effects on their perceived learning challenges in English listening and vocabulary. The qualitative results confirmed that listening, speaking, and writing were their three key challenging areas. Their specific learning challenges in each area were examined. Implications for Chinese college English teachers and administrators are discussed in terms of pedagogy and policy making, respectively.

## Introduction

English as a second or foreign language (ESL/EFL) education is a field that focuses on developing ESL/EFL students’ English language proficiency in the following eight areas: listening, speaking, reading, writing, pronunciation, grammar, vocabulary, and culture (Teaching English to Speakers of Other Languages [TESOL], 2017). Related research has shown that they experience considerable challenges in these areas and these challenges would eventually prevent them from developing their English language proficiency (Al-Mekhlafi and Nagaratnam, 2011; Shen, 2013; Chen and Lai, 2014; Yang and Chang, 2014; Boroujeni et al., 2015; Wang and Fan, 2015; Chang, 2016; Alhaysony and Alhaisoni, 2017).

China is a rapidly developing country with more EFL learners than any other countries in the world. English is taught and tested in all colleges and universities across the country (Liu and Huang, 2020; Zhao and Huang, 2020). There is no doubt that Chinese college students themselves play an extremely important role in their EFL learning; and they must be responsible for identifying their key challenges first and then developing strategies to cope with these challenges. Therefore, an examination of the key challenges in learning English from a Chinese college student perspective will provide important implications for Chinese college English teachers and college administrators/leaders in terms of EFL pedagogy and policy-making, respectively.

## A brief summary of the literature

Many empirical studies examined the challenges faced by EFL students in learning English (Liu and Jackson, 2008; Lovett et al., 2008; Zhang and Yin, 2009; Bao and Sun, 2010; Ma, 2012; Gan, 2013; Juan and Abidin, 2013; Rawlings and Sue, 2013; Shen, 2013; Chen and Lai, 2014; Liang, 2015; Ping et al., 2015; Wang and Fan, 2015; Alhaysony and Alhaisoni, 2017; Bawa and Watson, 2017; Sang, 2017). Specifically, they experience challenges in English listening, speaking, reading, and writing as well as English pronunciation, grammar, vocabulary, and culture (Liu and Jackson, 2008; Lovett et al., 2008; Zhang and Yin, 2009; Bao and Sun, 2010; Ma, 2012; Juan and Abidin, 2013; Chen and Lai, 2014; Sang, 2017). Since many empirical studies were conducted to examine the challenges faced by EFL students in each area, the researchers decided to choose two studies to review for each area.

### Challenges in English listening

Juan and Abidin (2013) found that the main problem faced by Chinese EFL students in English listening is the lack of prior knowledge of English vocabulary, which hinders their understanding in the listening process. In addition, the differences in native speakers’ accents prevent Chinese EFL students from correctly understanding the listening content. Similarly, Wang and Fan (2015) found that processing listening materials was difficult for low-proficiency Chinese learners. Moreover, accent and other varieties of standard English also pose difficulties to Chinese students, as they believe that these factors would increase their challenges in English listening.

### Challenges in English speaking

Liu and Jackson (2008) explored Chinese EFL students’ unwillingness to communicate and anxiety in English language classrooms and reported that many of them did not like to risk speaking English in class because they felt anxious in their English language classrooms. Further, by investigating EFL students’ oral English difficulties in China. Gan (2013) found that the perceived difficulties include language defects, oral process, conversational skills and academic speaking conventions, affective influences, and opportunities for students to use English for oral communication in learning contexts.

### Challenges in English reading

Lovett et al. (2008) explored whether struggling EFL readers from different primary language backgrounds differed in response to phonologically based remediation. The results indicated that syntactic awareness, and breadth and width of vocabulary were challenging for EFL readers. Further, Shen (2013) investigated technical university EFL learners’ academic reading difficulties, strategies, and learning needs. Most of the participants’ academic reading difficulties could be broadly attributed to deficiency in their language ability and their inability to comprehend content matter. Vocabulary was perceived to be the primary challenge when reading content-area textbooks.

### Challenges in English writing

Sang (2017) found that due to the negative impact of the curriculum and syllabus, high-risk standardized tests and the language environment of many Chinese universities, Chinese EFL students find it difficult to apply their English knowledge to writing. In addition, their English writing skills and strategies are also limited. Further, Bawa and Watson (2017) investigated the English writing challenges faced by Chinese EFL students in the United States and found that Chinese EFL students have serious English writing deficiencies due to their lack of language competence and the defects of the complex English language teaching system.

### Challenges in English pronunciation

Liang (2015) found that the challenge Chinese EFL students face in pronunciation is the influence of their mother tongue. Because of the negative influence of their mother tongue, their performance of pronunciation in making connected speech was poor. Similarly, Zhang and Yin (2009) reported similar results. They found that mother tongue is the first language interference to Chinese EFL students’ English pronunciation. In addition, Chinese EFL students’ lack of phonological and phonetics knowledge has greatly affected their pronunciation.

### Challenges in English grammar

Bao and Sun (2010) suggested that most Chinese EFL students believed that the challenge they faced in English grammar was that they could not remember grammar rules and could not use grammar rules correctly. In addition, students often make mistakes in the use of modal verbs, tense errors, verb noun confusion, and preposition mismatch or loss. Further, Alhaysony and Alhaisoni (2017) investigated grammatical difficulty from the perspective of Saudi Arabia EFL students as well as from the perspective of university teachers. The results showed that some English grammar features were more difficult and some were less difficult than others. For example, the five most difficult English grammar features reported in this study included unreal conditional, participial construction, real conditional, embedded question, and prepositions.

### Challenges in English vocabulary

Ma (2012) reported that Chinese EFL students have inadequate vocabulary learning strategies, low motivation, no collaborative learning environment to practice their lexical competence, insufficient exposure to authentic language input, and inadequate teachers’ instruction on social strategies. Further, Ping et al. (2015) investigated Chinese EFL learners’ needs in vocabulary learning. The results suggested that the students possessed insufficient knowledge of high-frequency words and were deficient in using cognitive deep processing strategies and metacognitive control strategies.

### Challenges in English culture

Two studies examined EFL students’ English culture challenges (Rawlings and Sue, 2013; Chen and Lai, 2014). In Rawlings and Sue’s (2013) study, the constructs of American culture and models of English language taught in Chinese classrooms are compared with the reality of American culture and English communication. It was found that Chinese students may often be unprepared for the challenges they will face, which are often associated with cultural dissimilarities and communicating in the English language. Further, Chen and Lai (2014) explored the influence of universality and specificity of culture on EFL learners’ comprehension of metaphor and metonymy. It was found that cultural differences may result in miscomprehension of metaphors and metonymy.

To sum up, the existing literature examined EFL learners’ challenges in different areas of learning English. However, these studies were based on small scale data, which could limit the generalization of research findings to a specific EFL learning context; furthermore, few studies examined these challenges from the perspective of EFL students. This national study aimed to move forward the research area and provide implications for pedagogy and policy making.

## Research purpose and questions

This mixed-methods national study has a twofold purpose: (a) to invite Chinese college students to rate their overall difficulties in the eight areas of English learning, i.e., listening, speaking, reading, writing, pronunciation, grammar, vocabulary, and culture; and (b) to invite them to identify their key challenges in learning EFL. Specifically, the following three research questions were asked: (a) What are their overall difficulties in the eight areas of learning EFL? (b) Are there significant differences in rating their overall difficulties in these eight areas across the demographic variables of gender (i.e., male versus female participants) and subject discipline (arts versus sciences versus English subject disciplines participants)? and (c) What are their key challenges in learning EFL?

## Materials and methods

The instrument of this study was a survey that consisted of a demographic information section which asked about participants’ gender and subject discipline, a five-point Likert scale rating form, and three essay questions of approximately 300 words each. The rating form invited participants to indicate their perceived difficulty level of each of the eight areas of English learning, i.e., listening, speaking, reading, writing, pronunciation, grammar, vocabulary, and culture on a five-point Likert scale ranging from 1 (least difficult) to 5 (most difficult). The first essay question required the participants to specifically describe their key challenges in learning English. The second essay question required them to describe their strategies to cope with these key challenges. The final essay question required them to describe their self-assessment practice in learning English as well as their perceived effectiveness and benefits of self-assessment (Mok et al., 2006; Earl, 2012). The qualitative data can help to probe deeply into the research setting to obtain in-depth understandings about Chinese college students’ English learning; and therefore, they can greatly enhance the quantitative data (Creswell, 2014).

The participants included a sample of 1,525 freshmen and sophomore students currently studying at seven universities across China. These seven universities were purposively identified for this study. The criteria for inclusion included (a) it must be a 4-year comprehensive university; (b) it must have more than 20,000 students on campus; and (c) it must have a school of English, where students learn English as their subject discipline. These seven universities are located in the eastern, southern, western, northern, and central parts of China, respectively. They were purposefully selected because they were considered a representative sample of all universities in China in terms of the numbers of schools and students on campus.

A total of 2,000 surveys were distributed to students in these universities, 1,529 completed ones were collected, with a response rate of 76.5%. Among these 1,529 collected surveys, four were considered invalid and excluded for the data analysis. Therefore, a total of 1,525 valid surveys were included in the final data analysis. In addition, these 1,525 participants come from 26 subject disciplines. For the ease of data analysis, these 26 subject disciplines were categorized into arts, sciences, and English subjects.

Among the 1,525 participants, 584 (38.3%) were male and 941 (61.7%) female participants; 514 (33.7%) were freshmen and 1,011 (66.3%) sophomore students; and 747 (49.0%), 528 (34.6%), and 250 (16.4%) were arts, sciences, and English subject students, respectively. These students came from different provinces of China and they had learned English for approximately 10 years at the time of data collection. It is important to note that their prior English learning experiences at elementary and secondary schools varied considerably. For example, some students who had learned English at urban schools might have native English speakers as their English teachers and therefore had more English language exposure than those who learned English at rural schools. For data analysis, participants were grouped by gender and subject discipline for the purpose of examining significant differences.

Data were collected with the assistance of a contact person in each participating university. This contact person was responsible for distributing and collecting the surveys and then shipping to the first author of this study. The participants were provided with information about the study, and they all understood that their participation voluntary and their responses were strictly confidential.

Using SPSS, the five-point Likert scale data were analyzed quantitatively at three different levels. First, the internal consistency reliability of the rating form was calculated. Second, the descriptive as well as group statistics (i.e., the mean and standard deviation) for each area of English was calculated. Third, gender-by*-*subject discipline (*2* × *3*) factorial ANOVAs were performed to examine significant gender, subject discipline, and gender-by-subject discipline interaction effects on their rating of the difficult levels of the eight areas of English learning. It is important to note that since the participants from the seven universities come from different parts of China and their English learning programs are similar, significant differences among schools were not expected; and therefore, demographic variable of school was not included in the quantitative data analysis.

Finally, the essay data were analyzed qualitatively. To ensure data integrity and consistency, the data were entered into an Excel spreadsheet by the first and third researchers who have rich experience in qualitative data analysis including coding. Following that, each essay was color-coded and sorted into different categories and subcategories by the first and third researchers (coders) independently first, and then collaboratively; and finally, conceptually similar content was discussed, grouped together, and then categorized according to the recurring themes. This process was to ensure inter-coder reliability of the qualitative data analysis. To enhance its validity, direct quotes from participants were also incorporated (Creswell, 2014).

## Results and discussion

This article reports the findings on participants’ key challenges in learning English. The findings on their coping strategies and self-assessment practice have been addressed in separate articles. In this section, the internal consistency reliability of the rating form and the inter-coder reliability of the qualitative data were reported first, followed by the presentation and discussion of Chinese college students’ overall difficulties in the eight areas of English learning (i.e., listening, speaking, reading, writing, pronunciation, grammar, vocabulary, and culture), the significant differences across gender and field of study variables, and their key challenges in learning EFL.

### The reliability of the rating form

Since Cronbach’s alpha is the most popular measure of internal consistency reliability, it was computed as an indication of the reliability of the rating form. Although the rating form consisted of only eight five-point Likert scale items, it has been shown to be reliable, with a Cronbach’s alpha reliability coefficient of 0.81.

### The inter-coder reliability of the qualitative data

Cohen’s kappa was calculated to assess the inter-coder reliability of the qualitative data. The obtained kappa of was 0.87, indicating that the inter-coder reliability of the quality data was satisfactory.

### Descriptive and group statistics

The descriptive and group statistics results are presented in Table 1. The descriptive statistics reports the mean and standard deviation for the difficulty level of each area of English learning.

**TABLE 1:** Descriptive and group statistics.

Category	Descriptive statistics			Group statistics							
	***N***	***M***	**SD**	**Gender**	***N***	***M***	**SD**	**Subject**	***N***	***M***	**SD**
Listening	1525	3.82	1.03	Male	584	3.84	1.08	Arts	747	3.93	1.05
				Female	941	3.82	1.00	Sciences	528	3.78	1.05
								English	250	3.65	0.92
Speaking	1525	3.64	1.08	Male	584	3.59	1.16	Arts	747	3.72	1.09
				Female	941	3.71	1.03	Sciences	528	3.52	1.11
								English	250	3.74	1.03
Reading	1525	3.02	0.90	Male	584	3.07	1.00	Arts	747	3.06	0.89
				Female	941	3.01	0.85	Sciences	528	3.09	0.94
								English	250	2.84	0.87
Writing	1525	3.38	0.94	Male	584	3.38	1.01	Arts	747	3.33	0.92
				Female	941	3.35	0.89	Sciences	528	3.41	0.99
								English	250	3.41	0.85
Pronunciation	1525	3.26	1.71	Male	584	3.30	2.43	Arts	747	3.27	2.18
				Female	941	3.21	1.08	Sciences	528	3.15	1.10
								English	250	3.40	1.11
Grammar	1525	3.33	1.05	Male	584	3.34	1.13	Arts	747	3.30	1.09
				Female	941	3.35	1.01	Sciences	528	3.41	1.09
								English	250	3.32	0.94
Vocabulary	1525	3.07	1.02	Male	584	3.10	1.15	Arts	747	2.96	1.03
				Female	941	3.05	0.99	Sciences	528	3.13	1.10
								English	250	3.21	0.98
Culture	1525	3.29	1.01	Male	584	3.31	1.20	Arts	747	3.27	1.11
				Female	941	3.28	1.01	Sciences	528	3.38	1.16
								English	250	3.17	0.81

As shown in Table 1, English listening (mean = 3.82), speaking (mean = 3.64), and writing (mean = 3.38) were identified as the most difficult areas for all the participants; whereas English reading (mean = 3.02), vocabulary (mean = 3.07), and pronunciation (mean = 3.26) the least difficult areas. These results confirmed previous research findings about Chinese students’ challenges in English listening (Yang and Chang, 2014; Wang and Fan, 2015), speaking (Liu and Jackson, 2008), and writing (Chang, 2016).

For male participants, English listening (mean = 3.84), speaking (mean = 3.59), and writing (mean = 3.38) were identified as the most difficult areas; whereas English reading (mean = 3.07), vocabulary (mean = 3.10), and pronunciation (mean = 3.30) the least challenging areas. For female participants, English listening (mean = 3.82), speaking (mean = 3.71), and writing (mean = 3.35) were identified as the most challenging areas; whereas English reading (mean = 3.01), vocabulary (mean = 3.05), and pronunciation (mean = 3.21) the least challenging areas.

For arts students, English listening (mean = 3.93), speaking (mean = 3.72), and writing (mean = 3.33) were identified as the most difficult areas; whereas English vocabulary (mean = 2.96), reading (mean = 3.06), and pronunciation (mean = 3.27) and culture (mean = 3.27) the least challenging areas. Similarly, for sciences students, English listening (mean = 3.78), speaking (mean = 3.52), and writing (mean = 3.41) were identified as the most challenging areas; whereas English reading (mean = 3.09), pronunciation (mean = 3.15), and vocabulary (mean = 3.13) the least challenging areas. However, for English subject discipline students, English speaking (mean = 3.74), listening (mean = 3.65), and writing (mean = 3.41) and pronunciation (mean = 3.40) were identified as the most challenging areas; whereas English reading (mean = 2.84), culture (mean = 3.17), and vocabulary (mean = 3.21) the least challenging areas.

### The gender-by-subject discipline factorial ANOVAs results

Eight gender-by-subject discipline (2 × 3) factorial ANOVAs were performed to examine the significant gender, subject discipline, and gender-by-subject discipline interaction effects on participants’ rating of the difficult levels of the eight areas of English learning. The 2 × 3 factorial ANOVAs for English speaking, reading, writing, pronunciation, grammar, and culture yielded no significant findings. However, the two-way factorial ANOVAs for English listening and vocabulary did yield significant findings. The results are presented in Table 2.

**TABLE 2:** The gender-by-subject discipline (2 × 3) factorial ANOVAs for English listening and vocabulary.

Area of English learning	Source	*df*	Mean square	*F*	Significant	Effect size
Listening	Gender	1	0.000	0.000	n.s.	0.000
	Subject	2	5.56	5.28	**	0.007
	Gender-by-subject	2	2.48	2.35	n.s.	0.003
Vocabulary	Gender	1	2.08	1.88	n.s.	0.001
	Subject	2	7.95	7.21	**	0.009
	Gender-by-subject	2	0.95	0.86	n.s.	0.001

**p < 0.01; n.s., not significant.

As shown in Table 2, the only significant main effect was found for participants’ subject discipline. The gender main effect and gender-by-subject discipline interaction effect were not significant. Specifically, English listening was identified as significantly more challenging for arts subject (mean = 3.93) students than for English subject (mean = 3.65) students (*p* < 0.01, effect size = 0.007). Further, English vocabulary was found to be significantly more difficult for sciences subject (mean = 3.13) students than for arts subject (mean = 2.96) students (*p* < 0.05, effect size = 0.009).

It is important to note that the obtained effect size was small for each of the two-way factorial ANOVAs which yielded significant group differences. Effect size identifies the strength of the conclusions about groups; and it often provides a more practical reading of the results (Creswell, 2014). The obtained small effect sizes for these ANOVAs suggest that these findings were significant only due the large sample size of this national study. Therefore, these significant differences should be interpreted with caution.

### Chinese college students’ key challenges in learning EFL

The essay question required all participants to describe their key challenges in learning EFL. Their essays were coded and then categorized into the following eight areas, i.e., English listening, speaking, writing, grammar, culture, pronunciation, vocabulary, and reading, in the order of their perceived levels of difficulty. The major findings are presented in Table 3.

**TABLE 3:** Chinese college students’ key challenges in learning English.

Category	Key challenges	Number of students	
		**Total valid**	**Total identified**
Listening	(a) A lack of authentic language exposure; (b) unfamiliarity with English culture; and (c) limited vocabulary	1,525	738 (48.4%)
Speaking	(a) A lack of English speaking environment; (b) L1 interference; and (c) limited speaking practice	1,525	598 (39.2%)
Writing	(a) A lack of logical thinking; (b) poor language control; and (c) unfamiliarity with the style of English written communication	1,525	419 (27.5%)
Grammar	(a) L1 interference; (b) complexity of English grammar; and (c) a lack of interest in English grammar	1,525	224 (14.7%)
Culture	(a) Limited access to the English culture; (b) Chinese culture transfer; and (c) limited culture teaching	1,525	189 (12.4%)
Pronunciation	(a) L1 negative transfer; (b) limited authentic input; and (c) limited practice in English pronunciation	1,525	178 (11.7%)
Vocabulary	(a) Difficulty in memorizing; (b) difficulty in understanding the meaning in context; and (c) difficulty in using while writing and speaking	1,525	126 (8.3%)
Reading	(a) Limited vocabulary; and (b) low reading rate	1,525	98 (6.4%)

### Listening challenges

Among the 1,525 participants, 738 (48.4%) of them identified English listening as their most difficult area. Their specific listening challenges include (a) a lack of authentic language exposure; (b) unfamiliarity with English culture; and (c) limited listening practice.

#### A lack of authentic language exposure

Among these 738 participants, approximately two-thirds of them reported that they did not have enough English language exposure while learning. The following are only a few comments made by some of them: “*My English teachers in the secondary schools rarely spoke English in the classroom*,” “*I did not receive much English listening practice while learning English in my high school*,” and “*to be honest, I had very limited authentic English listening materials when I was learning English*.”

#### Unfamiliarity with English culture

Among these 738 participants, about half of them reported that their unfamiliarity with English culture had become an obstacle to their English listening. “*I had great difficulty in comprehending listening materials with English cultural elements*,” and “*I could hardly understand the conversational implicatures because they are heavily associated with English culture*,” are a couple of selected comments made by the participants.

#### Limited vocabulary

Among the 738 participants, about one-third of them stated that their English listening challenges were caused by their limited English vocabulary. The following statements were selected from a few essays written by these participants. “*I could not make sense of what I was listening to because I did not know many words in the listening materials*,” “*I could not recognize many words and expressions while listening to English texts*,” and “*I always felt anxious in my English listening tests because of my limited English vocabulary.*”

Previous researchers such as Wang and Fan (2015), and Yang and Chang (2014) did report Chinese EFL students’ difficulties in English listening. They seemed to focus on specific listening challenges but this study on general listening challenges.

### Speaking challenges

Among the 1,525 participants, 598 (39.2%) of them reported English speaking as their key challenge in learning EFL. Their specific speaking challenges are (a) a lack of English speaking environment; (b) L1 interference; and (c) limited speaking practice.

#### A lack of English speaking environment

Among these 598 participants, approximately half of them reported that they did not have an English speaking environment while learning EFL. “*I could hardly find anyone who speaks English*,” “*there was no English speaking environment inside and outside the school*,” and “*learning English without speaking it could be disastrous to learning it as a foreign language*,” are a few selected comments made by these participants.

#### L1 interference

Again, among these 598 participants, about half of them mentioned that Chinese always interferes with their English speaking. There exists a direct translation process when they try to speak English. The following comment is a good explanation of this direct translation process in their English speaking. “*I cannot think in English, I always put everything in Chinese in my mind first, and then translate it into English, and finally speak in out in English*.” they did not have an English speaking environment while learning EFL.

#### Limited speaking practice

Among these 598 participants, about one-third of them commented that limited speaking practice makes their English speaking challenging and difficult. Many of them commented that their English teachers often focus on grammar and reading, without allotting much time for speaking in the classroom. Further, as one of them commented that, “*it is almost impossible to practice my English speaking outside the school*.”

Some of these challenges are similar to what Liu and Jackson (2008) had reported about Chinese EFL students’ speaking difficulties. The findings of this study did confirm that Chinese students face considerable challenges in their English speaking learning process.

### Writing challenges

Among the 1,525 participants, 419 (27.5%) of them reported writing as the most challenging area. Their specific writing challenges include (a) a lack of logical thinking; (b) poor language control; and (c) unfamiliarity with the style of English written communication.

#### A lack of logical thinking

Among these 419 participants, approximately 50% of them reported that they had challenges in organizing ideas logically in English writing. “*I feel difficult in writing a good paragraph*… *I know a topic sentence is important, but I have difficulty in stating the topic sentence properly*,” commented by one of the participants. “*I could not connect the paragraphs within an essay logically*,” added by another participant. Other participants also mentioned that they had difficulty in using the correct transition signals within an English essay.

#### Poor language control

Among these 419 participants, approximately more than 40% of them reported that they had poor language control in English writing. The following comments were selected from their essays: “*I usually could not write long English sentences*,” “*I feel difficult to pick up the right words and expressions in writing an English essay*,” and “*organizing ideas within a paragraph and an essay is challenging for me.*”

#### Unfamiliarity with the style of English written communication

Among these 419 participants, approximately one third of them reported that their unfamiliarity with the style of English written communication had become an obstacle to their English writing. They had noticed that the style of written communication was very different between English and Chinese. However, the style of Chinese written communication often interferes with their English writing. “*I usually follow the style of writing a Chinese essay when writing an English essay*,” “*I often fail to present my thesis statement at the beginning of an essay*,” and “*I use ‘always’ and ‘never’ frequently in my English writing*,” are just a few comments made by the participants.

Different from what Chang (2016) had reported about EFL doctoral students’ challenges in academic writing, this study examined Chinese college students’ challenges in English writing in general. The findings of this study would have important implications for EFL learning and teaching.

### Grammar challenges

Among the 1,525 participants, 224 (14.7%) of them reported grammar as the most challenging area. Their specific challenges in grammar are (a) L1 interference; (b) complexity of English grammar; and (c) a lack of interest in English grammar. Some of these results are similar to the research findings by Alhaysony and Alhaisoni (2017) and Al-Mekhlafi and Nagaratnam (2011).

#### L1 negative transfer

Among these 224 participants, approximately 45% of them reported that they Chinese grammar interferes with their English grammar learning. For example, they have difficulty in using English articles. One participant commented that “*the use of articles in front of nouns are required in English, but there is generally no such a requirement in Chinese grammar*… *I often miss the articles when I am writing in English*.” Some of them also mentioned that they had encountered great difficulty in English verb conjugation and using the correct tenses in their English writing and speaking.

#### Complexity of English grammar

Among these 224 participants, about 35% of them reported that the complexity of English grammar had made English grammar learning challenging and difficult. They listed quite a few grammar rules they felt complex and challenging, for example, the agreement between the subject and predicate of a sentence, the verb tenses and voices, the compound and complex sentences, the articles, the prepositions, and prepositional phrases Chinese grammar interferes with their English grammar learning.

#### A lack of interest in English grammar

Among these 224 participants, about 30% of them stated that they showed little interest in learning English grammar. The following are a few comments selected from their essays. “*I do not like to memorize the grammar rules*,” “*English grammar rules always come with exceptions, which made it hard for me to learning English grammar*… *therefore, I hate memorizing them [exceptions]*,” “*my English teachers spent a lot of time in the classroom teaching English grammar rules*… *we are eventually asked to memorize these [grammar] rules*,” and “*learning English grammar is boring*.”

### Culture challenges

Among the 1,525 participants, 189 (12.4%) of them reported that their most challenging area was related to English culture. Their specific culture challenges include (a) limited access to the English culture; (b) Chinese culture transfer; and (c) limited culture teaching. Some of these findings are in conformity with the previous literature (Rawlings and Sue, 2013; Chen and Lai, 2014).

#### Limited access to the English culture

Among these 189 participants, almost 80% of them reported that they had very limited access to the English culture when they learned English in the middle schools. The English textbook became their only access to the English culture. The following are only a few comments made by these participants: “*my understanding of the English culture came from the English textbooks,” “I did not usually watch English movies and TV programs while learning English in the middle schools,”* and *“I have never spoken to a native speaker of English and am not sure about what to talk about even if I have such an opportunity.”*

#### Chinese culture transfer

Among these 189 participants, about half of them reported that the Chinese culture definitely interfered with their learning of English and its culture. One participant made the following comment: “*I had encountered many difficulties in learning English speech acts, for example, how to respond to compliments. When someone said to me ‘your English is very good’ I would reply ‘my English is poor,’ which sounded very Chinese but awkward and inappropriate in English.*” The cultural difference between Chinese and English also created challenges for Chinese EFL learners. Many participants commented that they could not really understand the conversational implicatures and jokes when they spoke English with native speakers, which could make the conversations very embarrassing.

#### Limited culture teaching

Among these 189 participants, about 45% of them reported that their English teachers usually did not teach English culture in the classroom. The following are two comments made by these participants: “*my English teacher often focused on reading and grammar in the classroom without specifically touch English culture, to be very honest*,” and “*my [English] teacher chose to skip the teaching of [English] culture even the text contained some [English] cultural elements*.”

### Pronunciation challenges

Among the 1,525 participants, 178 (11.7%) of them identified English pronunciation as their most challenging area in their English learning. Their specific pronunciation challenges include (a) L1 negative transfer; (b) limited authentic input; and (c) limited practice in English pronunciation. These findings provide much more information about Chinese EFL students’ pronunciation challenges than the study conducted by Hui-Ling and Radant (2009).

#### L1 negative transfer

Among these 178 participants, approximately half of them reported that the Chinese sound system had a negative impact on their learning of English pronunciation. For example, several speech sounds such as (ae), (ð), (ts), and (dz) in the English sound system cannot be found in the Chinese sound system, which made the learning of these sounds difficult for Chinese students. Furthermore, other English phonetic features such as intonation, assimilation, and word and sentence stresses, as reported by most of them, caused them difficulty in learning English pronunciation because they are not available in the Chinese sound system.

#### Limited authentic input

Among these 178 participants, about 40% of them reported that they did not have sufficient authentic pronunciation materials while learning English pronunciation. The following is a typical comment made by one participant: “*I had great difficulty in learning English pronunciation due to the very limited authentic input. I am from a remote village where most people cannot speak standard Chinese. My three English teachers in the junior and senior middle schools had strong accents in speaking English. They spoke English differently and I could not tell who had the right pronunciation*.”

#### Limited practice in English pronunciation

Among the 178 participants, about 30% of them stated that they did not have much practice in English pronunciation. Since there are many differences between Chinese and English sound systems, practice becomes important for Chinese students to learn English pronunciation. The comment “*my [English] teachers’ focus was not on pronunciation in the classrooms and we had very limited practice in English pronunciation*” made by one participant became typical and common among the participants.

### Vocabulary challenges

Among the 1,525 participants, 126 (8.3%) of them identified English vocabulary as their most difficult area. Their specific vocabulary challenges include (a) difficulty in memorizing; (b) difficulty in understanding the meaning in context; and (c) difficulty in using while writing and speaking. These results confirmed the previous literature on Chinese EFL students’ challenges in English vocabulary (Ma, 2012; Ping et al., 2015).

#### Difficulty in memorizing

Among these 126 participants, approximately 90% of them complained that memorizing English words and expressions were extraordinarily difficult for them. They kept on forgetting the vocabulary they had remembered. One participant commented that “*when I was in the middle schools I tried to memorize [English] words every day but I also forgot them every day*… *I found memorizing English words tough*.” Several participants tried to find the reasons why they felt hard to memorize English words and made the following explanations. The two major reasons were (a) they usually did not know the pronunciation of each word they were trying to memorize and simply memorize its spelling by rote; and (b) they usually did not put the word in a specific context.

#### Difficulty in understanding the meaning in context

Among the 126 participants, about 70% of them mentioned that they felt difficult in understanding the meaning of a specific word or expression in context even if they knew its Chinese meaning. The following comment was made by one participant: “*I had difficulty in understanding the exact meanings of specific English words and phrases when I was reading a passage with these words and phrases in it. I knew I had remembered their Chinese meanings but I could not construct the meaning of a whole sentence or paragraph*.”

#### Difficulty in word use while writing and speaking

Among the 126 participants, about half of them commented that they felt difficult in using specific English words and expressions correctly when they were speaking and writing in English. Their biggest problem was the lexical collocations, which frequently occurred in their English speaking and writing. In addition, they had difficulty in using such linguistic units as set phrases, idioms, and sayings.

### Reading challenges

Among the 1,525 participants, only 98 (6.4%) of them considered reading as their most challenging area. Their specific reading challenges are (a) limited vocabulary; and (b) low reading rate. These findings are similar to what Shen (2013) reported about EFL college students’ reading challenges.

#### Limited vocabulary

Among these 98 participants, over 90% of them stated that their limited vocabulary caused them English reading comprehension challenges. With limited vocabulary, as indicated by many of them, they could not understand the meaning of each sentence in a passage, and eventually would not be able to comprehend the entire passage.

#### Low reading rate

Among the 98 participants, about half of them reported that their low reading rate caused them difficulty in English reading, The following comments were selected from their essays: “*I usually focused on every word when I was reading an English text, which made my reading rate really low*,” “*It took me forever to understand even 80% of the text I was reading*,” “*the reason behind it was probably because I read word by word, which made my reading comprehension rate low*,” and “*I realized that I did not have effective reading comprehension strategies and therefore my [English] reading rate was low*.”

## Conclusion

This article reported 1,525 Chinese college freshmen and sophomore students’ key challenges in learning EFL. The findings suggested that they confronted challenges in all eight areas of EFL learning, i.e., listening, speaking, reading, writing, pronunciation, grammar, vocabulary, and culture. Their three key challenging areas included listening (48.4%), speaking (39.2%), and writing (27.5%); whereas the bottom three areas are reading (6.4%), vocabulary (8.3%), and pronunciation (11.7%). In total, they reported 23 categories of key learning challenges across the eight areas of English learning (see Table 3).

This study was limited in the following two ways. First, this study was a qualitative method dominant empirical study; and investigated Chinese college students’ key challenges in their English learning from a general rather than specific perspective, which might limit the interpretation and generalization of the findings. Second, many other variables might have been included in this study about their past 10 years’ English learning such as where they came from, their past English test scores, and the specific methods they chose for learning English. However, the qualitative research in education generally involves participants’ self-reflections of their past learning (Mok et al., 2006; Earl, 2012) and it does not typically examine the effects of above mentioned variables provides a counter argument to this potential limitation.

In the light of these limitations, the following two conclusions were reached. First, these 1,525 Chinese college students did experience great challenges from all eight areas of EFL learning. Their challenges are not equally distributed across these areas. They experienced considerably more difficulties in English listening and speaking and much fewer difficulties in vocabulary and reading. Second, having an insufficient vocabulary size seemed to be a common problem across all four areas of listening, speaking, reading, and writing. Future research is needed to confirm and validate these findings.

The findings of this study provided pedagogical implications for Chinese college English teachers. For example, it is suggested that they consider adopting specific teaching methods to target students’ weakest areas such as listening, speaking, and writing. The findings of this study also offer policy making implications for Chinese college administrators and leaders. For example, it is suggested that they revise the existing policies or make new policies to either hire more native English teachers or create more opportunities for Chinese college students to study abroad for a period of time. These policies can surely meet students’ learning needs and help them achieve their English learning goals.

There are two possible directions for future research in this area. First, since the key challenges identified in this study were all from students’ perspective, future studies can be conducted to examine Chinese college English teachers’ comments on these challenges. Chinese teachers can also reflect on their own teaching and make adjustments accordingly. Second, future research is needed to conduct a quantitative method dominant study to examine Chinese college students’ key challenges in learning English from both student and teacher perspectives and at a more micro level so that the findings can be generalized to all other colleges and universities in China.

## Data availability statement

The raw data supporting the conclusions of this article will be made available by the authors, without undue reservation.

## Ethics statement

The studies involving human participants were reviewed and approved by the Ethics Review Committee of the Evidence-Based Research Center of Educational Assessment at Jiangsu University. The patients/participants provided their written informed consent to participate in this study.

## Author contributions

TS: literature research and review, data entry, preparation, and coding. YZ: conceptualization and data collection. All authors contributed to the article and approved the submitted version.
